# A Reminiscence of Lister

**Published:** 1912-02-24

**Authors:** L. K. Hodgkinson

**Affiliations:** Vicar. St. Peter's Vicarage, Upton, Essex


### The Editor's Letter Box.

### A REMINISCENCE OF LISTER.

*To the Editor of* The Hospital.

Sir,—I am writing in the room in which Lord Lister was born on April 5, 1827. The house is now the Vicarage and the Church of St. Peter is built upon a portion of the garden in which the great surgeon played as a boy. To the completion of this church Lord Lister himself contributed a few years ago, and we who worship in it think that it is a fitting home for a memorial to him, and that the best form that the memorial could take of one who did more than any other man has done to alleviate suffering and to save human life would be a rood screen with the figure of the Saviour of the World, and to this the character of the church is peculiarly adapted. We are all poor people here and cannot carry out our wish unaided. I therefore ask you of your courtesy to allow me to appeal through your columns to every one who has undergone an operation under antiseptic conditions to send me a donation for this purpose as a thank-offering for the benefit received, and to perpetuate, on the scene of his birth, the memory of one who has placed the whole world under an obligation.—I am, Sir, your obedient servant, L. K. Hodgkinson,

St. Peter's Vicarage, Vicar.

Upton, Essex, February 16, 1912.

